# Supplementary material for: Gene set enrichment analysis for genome-wide DNA methylation data
Source: Genome Biol. 2021 Jun 8;22:173. doi: 10.1186/s13059-021-02388-x (PMC8186068; doi:10.1186/s13059-021-02388-x)
Supplement: Supplementary file 3 — Additional file 3. Review history. [file 13059_2021_2388_MOESM3_ESM.docx]

Review History

**First round of review**

**Reviewer 1**

**Were you able to assess all statistics in the manuscript, including the appropriateness of statistical tests used?**

Yes: Yes, I have assessed the statistics and they seem fine to me

**Were you able to directly test the methods?**

No

**Comments to author:**

The paper "Gene set enrichment analysis for genome-wide DNA methylation data" by Belinda Phipson and colleagues presents a novel method for GSEA tailored for Illumina DNAm beadarray data. I fully agree with the authors that there is a need to build upon previous works to further develop improved methods for GSEA in this context, and which in particular avoid the biases associated with probe representation. The same authors previously presented a method called GSAmeth, which was published in Bioinformatics in 2016 and which was incorporated into the missMethyl R-package. In the current manuscript, the authors present an improvement called GOmeth, which has been incorporated into the same R-package, and which appears to have been motivated perhaps by the ebGSEA method which had been previously shown to outperform GSAmeth. However, the authors also extend their new method GOmeth to make it work on DMRs, which is entirely novel and an important practical advance. Overall, I think that the paper is well-written, but there are unfortunately also some concerns:

(1)	Whether the improvement GOmeth over the current state-of-the-art is an advance that merits publication in Genome Biology I am not sure, because the improvement if any is relatively marginal and not demonstrated across multiple datasets. For instance, panel 3D is nice, but this is just based on 1 dataset (normal kidney from TCGA). While the results shown in Fig.3D are robust to the number of samples, I would like to have seen more evidence for the same pattern in other datasets/contexts. I realize that picking real datasets and defining a ground truth in them maybe difficult or subject to misinterpretation, but it should be possible.

(2)	The difference between the new method GOmeth and the previous one GSAmeth is also not well articulated. It would appear that GOmeth deals with two intrinsic biases whereas GSAmeth dealt with one, correct? If so, this should made much clearer in the Introduction.

(3)	The statement on page-17 that ebGSEA can only be run with the MSigDB database is actually wrong. In fact, ebGSEA was published in Dong D et al Bioinformatics 2019, and in that paper there is a link to a github site with R-scripts that can be run completely independently from ChAMP. In fact, for various reasons, I would recommend the authors to run ebGSEA with other gene set databases using the R-scripts on that github site. It should be a relatively straightforward tweak to the author's code.

(4)	Related to the previous point, I am surprised that ebGSEA was excluded in Fig.4. Since it is possible to run ebGSEA with any gene-set database, I am puzzled why the authors chose not to display ebGSEA in this main figure. Could the authors perhaps add it to the figure, or explain why this is not possible?

(5)	Perhaps, the biggest concern I have is that although GOmeth appears to work well on the datasets analysed here, it is still dependent on a threshold……ie a threshold that determines the number of top-ranked CpGs to use. I am therefore a little worried that a general, possibly inexperienced, user, may not be able to make the right choice for their own datasets. While the authors provide some recommendations as to how many top-ranked CpGs to choose, this is very tricky. Therefore, I think that in Discussion, the authors should acknowledge this major limitation and that a more proper recommendation would be to advise users to use a *variety* of different methods. Indeed, the ebGSEA paper explicitly states this and I feel that a similar statement should be made in this manuscript, unless of course the authors can solve the threshold problem in some way that does not require a choice from the user. Or the authors would have to analyse many more datasets representing all different scenarios and make threshold recommendations for each scenario, which is probably unrealistic.

(6)	Overall, based on the data presented the real advance of the paper is the "regional" GOmeth, and therefore I would have expected the paper to be more focused on this particular aspect. Yet, this paper has 2 figures that display results with GOmeth, and 1 figure that displays results with the regional analysis. I am not sure if this is the right balance for this journal. I would recommend one additional figure and more analyses in support of GOregion.

**Reviewer 2**

**Were you able to assess all statistics in the manuscript, including the appropriateness of statistical tests used?**

Yes: I looked at these methods equations very briefly, so worth having another reviewer on this topic.

**Were you able to directly test the methods?**

No

**Comments to author:**

This is a well-written paper proposing new methods that address an important topic which is often done in methylation papers, but rarely using clear systematic approaches.

Overall the code, analysis, results are clear - the methods and paper are robust.

My main comment is that I wonder if co-methylation should be taken into account explicitly - ie in addition to probe-number bias? That is, if you have a gene with say 12 CpGs: 10 correlated CpGs and then another 2 further away that are not correlated with the each other or the rest - I feel that these 2 should have a greater weight than the 10 correlated ones. This could also be relevant in cases where CpGs annotated to different genes close together, which then also affects the region based analyses.

Minor comments

- It would be good to address again in the discussion the point that annotating CpGs to genes is not straighforward. Although most studies use the Illumina annotations, these can be somewhat of a black box, and some papers have used other methods e.g. in gene and within a specific distance to TSS.

- add a bit more in the results section on how probe number bias is handled in the new method and the weights

- Fig 3A,B and also relevant to D - include lines (or move current lines 1.5 over) to distinguish between sets of simulation scenarios into batches of 3 or 7

- Not sure that the immune sets are the best approach to assess performance of the method in distinguishing blood cell methylomes (page 10 lines 52-60). I feel that Figure 4D really addresses this point, as more specific immune pathways will be more relevant for different pair-wise cell-type comparisons. In my view the RNAseq data is the best 'truth' here to compare to (so form the main systematic evaluation), while the immune set analyses are interesting and give informative results, but are difficult to assess as a 'truth'.

**Authors Response**

**Point-by-point responses to the reviewers’ comments:**

*Response to reviewers:*

*We would like to thank the reviewers for their insightful reviews of our paper and for bringing up some interesting points to consider. We have addressed all the comments; in particular, we have analysed two new datasets, repeated our analysis using the new software for the ebGSEA methods and have incorporated these new results into our main and supplementary figures. We have added text in order to clarify some key points about our methods.*

Reviewer #1: The paper "Gene set enrichment analysis for genome-wide DNA methylation data" by Belinda Phipson and colleagues presents a novel method for GSEA tailored for Illumina DNAm beadarray data. I fully agree with the authors that there is a need to build upon previous works to further develop improved methods for GSEA in this context, and which in particular avoid the biases associated with probe representation. The same authors previously presented a method called GSAmeth, which was published in Bioinformatics in 2016 and which was incorporated into the missMethyl R-package. In the current manuscript, the authors present an improvement called GOmeth, which has been incorporated into the same R-package, and which appears to have been motivated perhaps by the ebGSEA method which had been previously shown to outperform GSAmeth. However, the authors also extend their new method GOmeth to make it work on DMRs, which is entirely novel and an important practical advance.

Overall, I think that the paper is well-written, but there are unfortunately also some concerns:

(1) Whether the improvement GOmeth over the current state-of-the-art is an advance that merits publication in Genome Biology I am not sure, because the improvement if any is relatively marginal and not demonstrated across multiple datasets. For instance, panel 3D is nice, but this is just based on 1 dataset (normal kidney from TCGA). While the results shown in Fig.3D are robust to the number of samples, I would like to have seen more evidence for the same pattern in other datasets/contexts. I realize that picking real datasets and defining a ground truth in them may be difficult or subject to misinterpretation, but it should be possible.

*We have added an additional dataset of normal samples from TCGA BRCA data (Supplementary figure 3C, D) and recreated Figure 3D using the updated ebGSEA functions from the GitHub repository. We have analysed an additional dataset studying B-cell development that had matched gene expression data that was appropriate for the generation of a gene set testing truth set. The methylation data was measured on 450K arrays and the gene expression was measured using Affymetrix arrays (PMID: 23074194). This “truth” set was then used to assess the methylation array gene set testing methods at the probe and region level (Supplementary Figures 4E, G, Supplementary Figures 8, 9, 14, 15).*

(2) The difference between the new method GOmeth and the previous one GSAmeth is also not well articulated. It would appear that GOmeth deals with two intrinsic biases whereas GSAmeth dealt with one, correct? If so, this should be made much clearer in the Introduction.

*We have modified the text in the introduction to clarify the improvements we have made to GOmeth.*

(3) The statement on page-17 that ebGSEA can only be run with the MSigDB database is actually wrong. In fact, ebGSEA was published in Dong D et al Bioinformatics 2019, and in that paper there is a link to a github site with R-scripts that can be run completely independently from ChAMP. In fact, for various reasons, I would recommend the authors to run ebGSEA with other gene set databases using the R-scripts on that github site. It should be a relatively straightforward tweak to the author's code.

*We appreciate the reviewer bringing this to our attention as we generally make the assumption that a Bioconductor package includes the most up-to-date methods while GitHub repositories typically house code that is still undergoing development. We also noticed that the ebGSEA R package on GitHub was only created on 3 June 2020, and we had already performed the majority of our evaluations by this date. We have now, however, repeated our analyses with the ebGSEA functions from the GitHub repository and updated our figures accordingly. We have also removed Supplementary Figures 8, 9, 10 as they were only included to allow comparison to the ChAMP implementation of ebGSEA, using the built-in MSigDB gene sets.*

(4) Related to the previous point, I am surprised that ebGSEA was excluded in Fig.4. Since it is possible to run ebGSEA with any gene-set database, I am puzzled why the authors chose not to display ebGSEA in this main figure. Could the authors perhaps add it to the figure, or explain why this is not possible?

*We believe our previous comment answers this question. We have now added the ebGSEA methods to Figure 4 and Supplementary Figures 5, 6 and 7.*

(5) Perhaps, the biggest concern I have is that although GOmeth appears to work well on the datasets analysed here, it is still dependent on a threshold……ie a threshold that determines the number of top-ranked CpGs to use. I am therefore a little worried that a general, possibly inexperienced, user, may not be able to make the right choice for their own datasets. While the authors provide some recommendations as to how many top-ranked CpGs to choose, this is very tricky. Therefore, I think that in Discussion, the authors should acknowledge this major limitation and that a more proper recommendation would be to advise users to use a *variety* of different methods. Indeed, the ebGSEA paper explicitly states this and I feel that a similar statement should be made in this manuscript, unless of course the authors can solve the threshold problem in some way that does not require a choice from the user. Or the authors would have to analyse many more datasets representing all different scenarios and make threshold recommendations for each scenario, which is probably unrealistic.

*We appreciate the reviewer’s point regarding setting a threshold, and indeed this is always required with methods such as GOmeth/GOregion that are based on a hypergeometric test, or other methods that use Fisher type tests (which is common for GSEA methods for gene expression data, e.g. DAVID, ToppFun, or the Bioconductor package GOstats). This is an important decision, and one we feel that users should be aware of, hence why we have now included this as Discussion in our paper. We would hope that by highlighting this point we can empower users to better understand their data and the accompanying statistical tests they are using for gene set enrichment analysis. We personally feel that allowing the user to have flexibility with regards to the significant CpGs/regions that are used as input to a gene set test is preferable to a black box approach where this threshold is arbitrarily set within the software, as this will not be suitable for all datasets. Hence we have recommended that for datasets with tens of thousands of significant CpGs, GOregion is likely to produce the most biologically relevant results, and that in general no more than 10,000 significant CpGs should be used as input to GOmeth. However, we do appreciate the reviewer’s point of view, and have added some sentences to our discussion recommending the use of mGLM and ebGSEA as alternative methods that perform well in the context of a probe-wise analysis and do not require setting a threshold cut-off.*

(6) Overall, based on the data presented the real advance of the paper is the "regional" GOmeth, and therefore I would have expected the paper to be more focused on this particular aspect. Yet, this paper has 2 figures that display results with GOmeth, and 1 figure that displays results with the regional analysis. I am not sure if this is the right balance for this journal. I would recommend one additional figure and more analyses in support of GOregion.

*GOregion builds on the methodology of GOmeth, which is why we thought it was important to thoroughly evaluate GOmeth to be satisfied that it was performing as expected. We have added the analysis of an additional dataset using GOregion (new Supplementary Figures 14 and 15).*

Reviewer #2: This is a well-written paper proposing new methods that address an important topic which is often done in methylation papers, but rarely using clear systematic approaches.

Overall the code, analysis, results are clear - the methods and paper are robust.

*Thank you!*

My main comment is that I wonder if co-methylation should be taken into account explicitly - ie in addition to probe-number bias? That is, if you have a gene with say 12 CpGs: 10 correlated CpGs and then another 2 further away that are not correlated with each other or the rest - I feel that these 2 should have a greater weight than the 10 correlated ones. This could also be relevant in cases where CpGs annotated to different genes close together, which then also affects the region based analyses.

*This is a really interesting point and one that we had not explicitly addressed with regards to how we accounted for the biases in gene set testing for the 450K and EPIC arrays. We understand that correlated CpGs will not add independent information about whether a gene is differentially methylated, and that co-methylation could be used to modify equivalent numbers of CpGs in a gene. This would potentially change the probability weighting function and hence the probability of differential methylation for each gene.*

*However:*

*(1) Rather than a technical effect (like multi-mapping probes), co-methylation is really a biological phenomenon and it would be difficult for us to account for this directly without making strong assumptions regarding the biological interpretation. For example, is a CpG in a CpG island more biologically relevant, but also more likely to be correlated with other CpGs compared with a CpG in the body of a gene? This is part of the reason why region analysis has been so successful.*

*(2) It is difficult to actually measure the correlation of CpGs, especially if it is sample dependent. This would require a significant change to our current implementation and we feel that it is beyond the scope of the paper.*

*However, the current manner in which we calculate the probability weighting function, which is scale-invariant, is likely to take co-methylation into account in a broader sense. For example, we find that genes with more CpGs are more likely to have CpGs that are closer together, which is highly consistent for both the 450K and EPIC arrays (Figure 1). Because the probability weighting function is an overall trend across the number of CpGs per gene and is empirically derived for each dataset, it will naturally correct for this added trend in distance between CpGs as the number of CpGs increases. Thus, while we have been unable to address this interesting point directly in the software we feel that it is somewhat robust to this issue.*

*A final point that indirectly addresses this issue is that we have now implemented a parameter that allows the significant CpGs to be filtered for specific regions, such as promoters, which are often considered to be more functionally related to gene expression. We note that the distribution of probes in and around genes is quite biased towards being in the promoter for the 450K and EPIC arrays (Pidsley et. al., 2016). However, if a user was particularly interested in differentially methylated probes in areas of the gene other than the promoter, such as the gene body, this is an option that we have already implemented.*

*Figure 1: The relationship between the number of CpGs per gene and the distance between sequential probes per gene. For each gene, the median distance between adjacent CpGs was calculated, as well as the number of probes across each gene. The genes are grouped into bins containing 200 genes, ordered by nCpGs. For each bin, the average number of probes and the average median distance was calculated. The data is plotted on the log scale.*

*Reference:*

*Pidsley, R., Zotenko, E., Peters, T.J. et al. Critical evaluation of the Illumina MethylationEPIC BeadChip microarray for whole-genome DNA methylation profiling. Genome Biol 17, 208 (2016). https://doi.org/10.1186/s13059-016-1066-1*

Minor comments

- It would be good to address again in the discussion the point that annotating CpGs to genes is not straightforward. Although most studies use the Illumina annotations, these can be somewhat of a black box, and some papers have used other methods e.g. in gene and within a specific distance to TSS.

*We have added some text regarding this point following the first paragraph of Discussion.*

- add a bit more in the results section on how probe number bias is handled in the new method and the weights

*We have rewritten the results section to better convey how probe-number and multi-gene bias is handled and the role of the weights. We have also tried to clarify the details of the statistical framework in ‘Methods’.*

- Fig 3A,B and also relevant to D - include lines (or move current lines 1.5 over) to distinguish between sets of simulation scenarios into batches of 3 or 7

*Done.*

- Not sure that the immune sets are the best approach to assess performance of the method in distinguishing blood cell methylomes (page 10 lines 52-60). I feel that Figure 4D really addresses this point, as more specific immune pathways will be more relevant for different pair-wise cell-type comparisons. In my view the RNAseq data is the best 'truth' here to compare to (so form the main systematic evaluation), while the immune set analyses are interesting and give informative results, but are difficult to assess as a 'truth'.

*Generally it is difficult to define any “truth” gene sets that we would expect to see in a real (and not simulated) dataset with methylation differences. However, we felt that by presenting both “truths”, as well as the top 10 most highly ranked gene sets from each method, we would hopefully be presenting a relatively unbiased evaluation of the methods rather than focussing on one “truth” set over the other. We have included this point in the Discussion to be transparent about the shortcomings of both “truth” sets. The “immune sets” will certainly capture the broad GO terms relating to blood and may not be specific to differences in blood cell types such as B-cells vs NK cells, whereas the RNA-Seq “truth” set may capture more specific cell type differences. However, the RNA-Seq “truth” set is not perfect either, as many experiments have shown that there is often only minor correlation between methylation and gene expression. Furthermore, the value of the dataset depends on the quality of the RNA-Seq/expression dataset and the methods used in the statistical analysis. Also, we only used the top 100 gene sets as our “truth set” and this may have resulted in us missing some true gene sets from our “truth”. Finally, we are relying heavily on the assumption that the gene expression and methylation data measure the activity of the same biological processes, and in the case of sorted cell types, this assumption is probably ok. However, for other types of biological systems the link between gene expression and methylation changes may not be as strong.*

**Second round of review**

**Reviewer 1**

The authors have done an excellent job in responding to my comments, and I do think that

the revised version is much improved. I only have one minor but essential request for revision:

- I agree that reporting the computational runtimes of algorithms is extremely important and I think that Table-1 is informative. However, the runtimes quoted are for 1-core. Nowadays, most groups have access to servers, or even a standard Dell workstation can be run with 20 or so cores. Given that there is ample space to add another column to Table-1, I would ask the authors to quote the runtimes for say a 20-core machine. This can be approximate, ie. not all operations within an algorithm may be parallelizable, but under the assumption that they are parallelizable, and that the effect would be linear, I would suggest that the authors list the estimated runtimes for a 20-core machine in a 2nd column in the same table. This is to make it clear that none of the methods presents a "limitation" as far as computational runtimes are concerned.

Looking forward to seeing this published.
